# Safety and Feasibility of Esophagectomy Following Neoadjuvant Immunotherapy Combined with Chemotherapy for Esophageal Squamous Cell Carcinoma

**DOI:** 10.3389/fsurg.2022.851745

**Published:** 2022-05-26

**Authors:** Yi-Min Gu, Qi-Xin Shang, Han-Lu Zhang, Yu-Shang Yang, Wen-Ping Wang, Yong Yuan, Yang Hu, Guo-Wei Che, Long-Qi Chen

**Affiliations:** Department of Thoracic Surgery, West China Hospital of Sichuan University, Chengdu, China

**Keywords:** safety, feasibility, esophagectomy, immune checkpoint inhibitor, neoadjuvant treatment

## Abstract

**Background:**

This study aimed to investigate the safety and feasibility of esophagectomy after neoadjuvant immunotherapy and chemotherapy for esophageal squamous cell carcinoma.

**Methods:**

We retrospectively identified patients who received neoadjuvant immunotherapy combined with chemotherapy (*n* = 38) in our center between 2020 and 2021. The primary end point was the risk of major complications (grade ≥3) according to the Clavien–Dindo classification. Secondary end points were surgical details, 30-day mortality, and 30-day readministration.

**Results:**

The most commonly used regimens of immunotherapy were camrelizumab (36.8%), pembrolizumab (31.5%), tislelizumab (15.8%), sintilimab (13.2%), and toripalimab (2.6%). The median interval to surgery was 63 days (range, 40–147). Esophagectomy was performed in 37 of 38 patients who received neoadjuvant immunotherapy and chemotherapy. All procedures were performed minimally invasively, except for 1 patient who was converted to thoracotomy. Of 37 surgical patients, R0 resection was achieved in 36 patients (97.3%). Pathologic complete response was observed in 9 patients (24.3%). Tumor regression grade I was identified in 17 patients (45.9%). Morbidity occurred in 12 of 37 patients (32.4%). The most common complication was pneumonia (16.2%). There were no deaths or readministration within 30 days.

**Conclusions:**

Esophagectomy following neoadjuvant immune checkpoint inhibitor plus chemotherapy for patients with resectable esophageal squamous cell carcinoma appears to be safe and feasible, with acceptable complication rates.

## Introduction

Esophageal cancer is the seventh most common malignant tumor and the sixth leading cause of cancer-related deaths worldwide (1). To date, neoadjuvant chemoradiotherapy plus surgery has formed the standard treatment for local advanced esophageal cancer based on the CROSS study and the NEOCRTEC5010 study (2, 3). However, approximately 29% of patients who underwent resection after chemoradiotherapy had a pathological complete response (2). The long-term outcomes of the CROSS study showed that 49% of patients had overall disease progression in the neoadjuvant chemoradiotherapy plus surgery group (4). In addition, the incidence of grade 3 or 4 treatment-related adverse events during chemoradiotherapy is common, such as leukopenia, anorexia, and fatigue (3). Radiation can also lead to normal tissue complications, including radiation-induced pneumonitis or esophagitis (5).

Immune checkpoint inhibitors (ICIs) have recently been explored as a novel strategy for improving the survival outcomes of esophageal cancer patients. ICIs, such as pembrolizumab, nivolumab, and camrelizumab, are indicated for the treatment of metastatic or locally advanced esophageal squamous cell carcinoma (ESCC) refractory to previous therapy (6–8). The survival benefit from adjuvant nivolumab in patients with resected esophageal cancer who had received neoadjuvant chemoradiotherapy was also confirmed according to CheckMate 577 (9). Therefore, the use of ICIs as neoadjuvant therapy for patients with ESCC has received substantial research attention. An increasing number of patients treated with preoperative chemotherapy plus ICIs need to accept operations. The safety and feasibility of esophagectomy in this setting remain unclear. The aim of this study was to analyze the clinical data, perioperative outcomes, and oncologic outcome data of ICIs plus chemotherapy as a neoadjuvant therapy regimen for the treatment of patients with locally advanced ESCC in our institution. These short-term outcomes may be helpful for future trials and surgical practice.

## Materials and Methods

### Study Design

We conducted a retrospective review of our prospectively collected database to identify patients who underwent esophagectomy within 6 months of treatment with ICIs combined with chemotherapy in our department between January 2020 and April 2021. The primary objective was to investigate the safety of esophagectomy after neoadjuvant immunotherapy and chemotherapy. The primary outcome was postoperative complications. The feasibility was determined as the proportion of patients able to undergo esophagectomy after neoadjuvant immunotherapy and chemotherapy.

This study was approved by the Ethics Committee of West China Hospital of Sichuan University on 15 June 2021 (No. 2021771). Informed consent was waived.

### Patient Eligibility

Inclusion criteria included (1) esophageal squamous cell carcinoma patients who underwent esophagectomy within 6 months of treatment with ICIs combined with chemotherapy; (2) ECOG performance status 0–2; (3) clinical TNM stage as T1N_+_M0 or T2–4aN0–3M0, diagnosed by means of esophagoscopy, contrast-enhanced computed tomography, ultrasonography, and positron emission tomography-computed tomography; and (4) measurable disease at baseline on the basis of Response Evaluation Criteria in Solid Tumors, version 1.1 (RECIST v 1.1).

Exclusion criteria included (1) coexistence of other malignancies; (2) autoimmune disease; and (3) radiation therapy.

### Treatment Protocol

All patients received neoadjuvant chemotherapy, and the recommended regimens included paclitaxel (175 mg/m^2^ i.v., d1, q3w) plus cisplatin (75 mg/m^2^ i.v., d1, q3w). Five PD-(L)1 inhibitors, pembrolizumab (2 mg/kg, i.v., q3w), tislelizumab (200 mg, i.v., q3w), camrelizumab (200 mg, i.v., q3w), sintilimab (200 mg, i.v., q3w) and toripalimab (3 mg/kg, i.v., q2w), were applied for neoadjuvant immunotherapy. The adverse event caused by neoadjuvant treatment was judged by the US National Cancer Institute’s Common Terminology Criteria for Adverse Events (CTCAE), Version 4.0 (10). A grade scale of grade 1 through 5 was provided for the description of severity for each adverse event.

After 2–4 cycles of neoadjuvant regimens, ESCC patients were recommended to undergo a preoperative assessment to determine the feasibility of the operation. All patients underwent minimally invasive McKeown esophagectomy. Cervical esophagogastrostomy was performed using hand-sewn double layer sutures. Two-field lymph node dissections were conducted.

### Data Analysis

We classified tumor stage according to the 8th edition of the TNM staging system of esophageal cancer (11). Postoperative complications after esophagectomy were defined according to the Esophagectomy Complications Consensus Group system and were graded according to the Clavien–Dindo classification (12, 13). Computed tomography or magnetic resonance imaging was performed at baseline and after treatment. The response of the primary lesion was also assessed according to imaging measures on the basis of RECIST v1.1. Pathologic complete response was defined as no residual disease in the primary tumor and regional lymph nodes. Tumor regression grade (TRG) was defined according to the Mandard system (14).

The characteristics of the patients were summarized in descriptive statistics. Normally distributed continuous variables are presented as the mean ± standard deviation (SD), nonnormally distributed continuous variables as the median with interquartile range (IQR), and categorical variables as frequencies and percentages. Statistical calculations were performed using SPSS Statistics (version 24, IBM, Armonk, NY).

## Results

**Figure 1:**
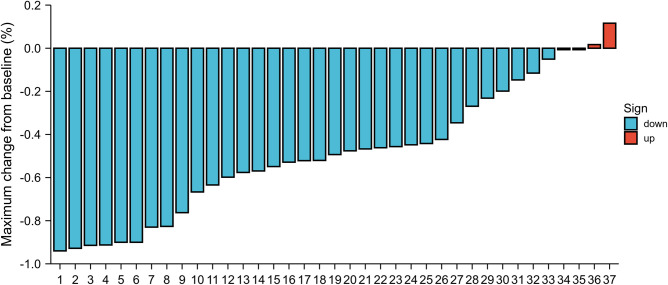
The best change in the longest primary lesion diameters from baseline.

The database query identified 38 consecutive patients with esophageal cancers who received ICIs therapy and chemotherapy in our hospital between January 2020 and April 2021. Demographic and clinical information for the cohort is listed in Table 1. The median age was 66 years (range, 46–80 years). Of 27 participants (71%) were men, and 11 participants (28%) were women. The majority of enrolled patients were clinical stage III. There was clinical stage II disease in 9 patients (23.7%); 29 patients (76.3%) had stage III disease. The most commonly used single-agent ICIs were camrelizumab (*n* = 14, 36.8%), pembrolizumab (*n* = 12, 31.5%), tislelizumab (*n* = 6, 15.8%), sintilimab (*n* = 5, 13.2%), and toripalimab (*n* = 1, 2.6%). In total, 7 patients (18.4%) experienced any grade adverse events in neoadjuvant therapy. The incidence of adverse events included the following: neutropenia (*n* = 4, 10.8%); fatigue (*n* = 3, 7.9%); hypothyroidism (*n* = 2, 5.3%); thrombocytopenia (*n* = 2, 5.3%); and elevated serum alanine aminotransferase or aspartate aminotransferase (*n* = 2, 5.3%). Other adverse events less than 5% included dermatitis, diarrhea and anorexia. Apart from 1 patient who had grade 3 dermatitis, there were no other grade 3 toxicities due to immunotherapy. The size of the primary esophageal lesion decreased in 33 (89.2%) patients by imaging assessment (Figure 1).

**Table 1:** Patient characteristics (*n* = 38).

Characteristic	Value
Median age (range), year	66 (46–80)
Sex, *n* (%)	
Male	27 (71.1)
Female	11 (28.9)
ECOG performance-status score, *n* (%)	
0	29 (76.3)
1	7 (18.4)
2	2 (5.3)
% predicted FEV1, median (range)	79 (58–93)
% predicted DLCO, median (range)	90 (46–121)
Pulmonary comorbidity, *n* (%)	9 (23.7)
Cardiac comorbidity, *n* (%)	7 (18.4)
Diabetes, *n* (%)	5 (13.2)
Smoking history, *n* (%)	16 (42.1)
cT	
2	3 (7.9)
3	35 (92.1)
cN	
0	9 (23.7)
1	5 (13.2)
2	24 (63.2)
cStage	
II	9 (23.7)
III	29 (76.3)
Regimen	
Camrelizumab	14 (36.8)
Pembrolizumab	12 (31.5)
Tislelizumab	6 (15.8)
Sintilimab	5 (13.2)
Toripalimab	1 (2.6)

*cT, clinical T stage; cN, clinical N stage (according to AJCC 8th edition); FEV1, forced expiratory volume in 1 s; DLCO, diffusing capacity for carbon monoxide*.

Surgical details and pathologic outcomes are summarized in Table 2. Of 38 patients who received neoadjuvant immunotherapy and chemotherapy, one patient had disease progression and was deemed unsuitable for surgical resection. The median duration from final treatment to surgery was 63 days (range, 40–147 days). Minimally invasive esophagectomy was performed in 37 patients. Of these, 1 patient was converted to thoracotomy. The median operative time was 260 min (range, 210–360 min). The median estimated blood loss was 100 mL (range, 20–200 mL). The median chest tube duration was 9 days (range, 5–64), and the median hospital length of stay was 11 days (range, 6–177).

**Table 2:** Perioperative outcomes of patients undergoing esophagectomy (*n* = 37).

Perioperative detail	Value
Interval to surgery, day, median (range)	63 (40–147)
Conversion (VATS converted to throcotomy), *n* (%)	1 (2.7)
Operative time, minutes, median (range)	260 (210–360)
Estimated blood loss, mL, median (range)	100 (20–200)
Surgical margins, *n* (%)	
R0	36 (97.3)
R1	1 (2.7)
No. of lymph node resected, median (range)	26 (10–75)
Chest tube duration, day, median (range)	9 (5–64)
Length of stay, day, median (range)	11 (6–177)
30-day mortality	0
30-day readmission	0
Pathologic Stage	
Pathologic complete response	9 (24.3)
IA	2 (5.4)
IB	3 (8.1)
IIA	2 (5.4)
IIB	8 (21.6)
IIIA	3 (8.1)
IIIB	6 (16.2)
IVA	2 (5.4)
Undetermined^a^	2 (5.4)
TRG	
1	17 (45.9)
2	8 (21.6)
3	7 (18.9)
4	5 (13.5)

^a^ *Two subjects with undetermined pathologic stage due to tumor response: pT0N2; pT0N1; TRG, tumor regression grade according to the Mandard system; VATS, video-assisted thoracoscopic surgery*.

**Figure 2:**
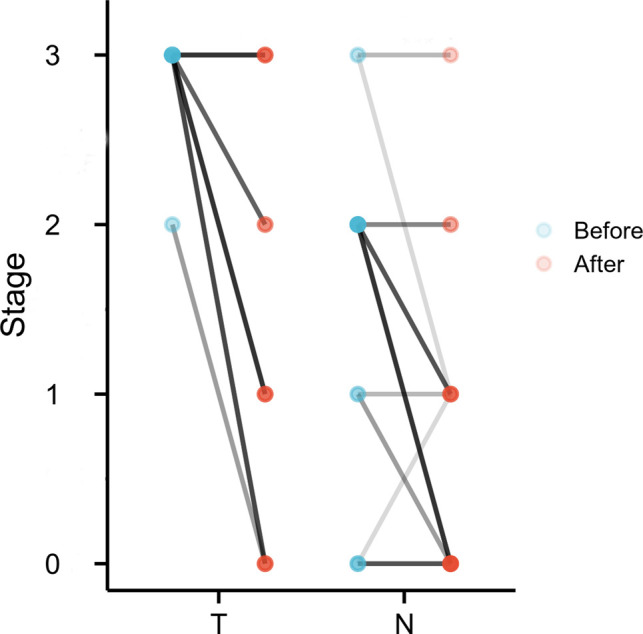
Change in T stage and N stage before and after treatment. Colors of lines, from light to dark, reflect the number of patients in which stage was correlated.

Of 37 surgical patients, 9 patients (24.3%) had a pathologic complete response; the pathologic stage of 2 patients (pretreatment cT3N2 and cT2N2) was undetermined because they had no residual viable tumor but were lymph node positive on the final pathologic assessment. In terms of clinical and pathologic tumor staging, 27 of 37 operative patients had tumor downstaging (73.0%), and 10 patients (27.0%) had no change in T status after surgery (Figure 2). TRG I was observed in 17 patients (45.9%). Of surgical patients, R0 resection was achieved in 36 cases (97.3%); 1 patient (2.8%) underwent incomplete (R1) resection. This was a patient with confluent metastatic coeliac lymph nodes invading the pancreas. The median number of lymph nodes resected was 26 (range, 10–75). In terms of clinical and pathologic nodal staging, 21 of the 37 surgical patients had nodal downstaging (56.8%); 12 (57.1%) of these patients had complete nodal clearance of disease. Fifteen patients (40.5%) had no change in N status after surgery, but 7 patients (46.7%) were clinically nodal negative. Notably, 1 patient (2.7%) had nodal upstaging (Figure 2).

The postoperative complications are reported in Table 3. In total, 12 patients (32.4%) experienced some postoperative complications. The most common any-grade postoperative complication was pneumonia in 6 patients (16.2%), pleural effusion in 3 patients (8.1%), anastomotic leakage in 2 patients (5.4%), and recurrent laryngeal nerve palsy in 2 patients (5.4%). Others with an incidence less than 5% included pneumothorax, respiratory insufficiency, cardiac arrhythmia, delayed gastric emptying, and chyle leakage. Regarding grade 3 or 4 complications, the most common were pneumonia in 2 patients (5.4%), pleural effusion in 2 patients (5.4%); anastomotic leakage in 2 patients (5.4%); pneumothorax in 1 patient (2.7%); respiratory insufficiency in 1 patient (2.7%); cardiac arrhythmia in 1 patient (2.7%); delayed gastric emptying in 1 patient (2.7%); and chyle leakage in 1 patient (2.7%). There was no 30-day readmission. No patients died within 30-day of surgery.

**Table 3:** Thirty-day complications (*n* = 37).

Event	Number of patients with event (percent)	
	Any grade	Grade 3 or 4
Pulmonary		
Pneumonia	6 (16.2)	2 (5.4)
Pleural effusion	3 (8.1)	2 (5.4)
Pneumothorax	1 (2.7)	0
Respiratory insufficiency	1 (2.7)	1 (2.7)
Cardiac		
Myocardial infarction	0	0
Cardiac arrhythmia	1 (2.7)	0
Anastomotic		
Anastomotic Leakage	2 (5.4)	2 (5.4)
Delayed gastric emptying	1 (2.7)	0
Other complications		
Bleeding	0	0
Wound infection	0	0
Recurrent laryngeal nerve palsy	2 (5.4)	0
Chyle leak	1 (2.7)	1 (2.7)

## Discussion

The improved overall survival and acceptable toxicity of chemotherapy combined with ICIs in patients with locally advanced or metastatic esophageal cancer (6–8) have led to many ongoing clinical trials of immunotherapeutic agents in the neoadjuvant setting. A summary of the perioperative outcomes might be critical to investigate the safety and feasibility of esophagectomy. Such data would be helpful for future trials and surgical practice.

To date, few studies have reported the safety and feasibility of esophagectomy following neoadjuvant immunotherapy combined with chemotherapy. In this study, esophagectomy in 37 patients with stage II to IVa ESCC after neoadjuvant immunotherapy combined with chemotherapy did not result in unexpected mortality. Conversion to thoracotomy appeared in one case, which appeared to be related to the inflammatory response and fibrosis at the primary tumor and involved nodal stations, presumably related to the treatment response. Several studies have reported that neoadjuvant immunotherapy may increase surgical difficulty in pulmonary resection (15, 16). Except for 1 patient who had grade 3 dermatitis, we observed almost no serious events due to immunotherapy. The proportion of patients able to undergo esophagectomy after neoadjuvant immunotherapy and chemotherapy was 97.4% (37 of 38 patients). Only 1 patient (2.6%) was unable to undergo esophagectomy because of the increased primary tumor size. The median operative time was 260 minu (range, 210–360 min), which is no longer than other studies (17, 18).

In this study, 9 patients (24.3%) experienced a pathologic complete response. This is less favorable than the 39.2% pathologic complete response rate in the NICE study of neoadjuvant immunotherapy combined with chemoradiotherapy (19). This might be attributed to varied treatment regimens or limited sample size. Many studies have pointed out that complete response was associated with significantly improved long-term survival compared with partial response (20, 21). However, Pamela et al. found that although neoadjuvant chemoradiotherapy more easily induced a complete response before esophagectomy, it was not an independent prognostic factor affecting long-term survival (22). Other factors impacting survival among complete responders and partial response patients deserve further investigation.

The most common complication in this study was pneumonia, which occurred in 6 patients (16.2%). This rate is lower than the 30% to 46% in the neoadjuvant chemoradiotherapy setting in other studies (2, 17). Serious postoperative complications were noted in 5.4% of patients. Other serious complications (more than 5%) included pleural effusion (5.4%) and anastomotic leakage (5.4%). Overall, the rates of perioperative complications were not higher than those in the neoadjuvant chemoradiotherapy setting (2, 17). Christophe et al. emphasized that neoadjuvant chemoradiotherapy increases postoperative mortality in patients with early-stage esophageal cancer compared with surgery alone (23). Based on the JCOG9907 trial (24), neoadjuvant doublet chemotherapy with cisplatin plus 5-fluorouracil was recommended as the standard treatment for patients with resectable squamous cell carcinoma in Japan. Perioperative complications were increased by adding radiation therapy compared to the perioperative chemotherapy group (25). However, patients in the chemoradiotherapy group might have higher pathologic complete response rates at resection. To date, no published study has compared the outcomes of neoadjuvant chemotherapy plus immunotherapy with neoadjuvant chemoradiotherapy in esophageal cancer.

As the use of ICIs is widespread in clinical practice, it is likely that surgeons will be asked to perform esophagectomy on increasing numbers of patients who have received immunotherapy. It is incumbent upon surgeons to assess perioperative outcomes under the novel strategy. This study represents the largest experience to date of esophagectomy after neoadjuvant ICIs combined with chemotherapy in patients with ESCC. There are also some limitations in this study. First, the inherent limitation is its retrospective nature. Next, the sample size was limited. Furthermore, only data on short-term outcomes were available, and the long-term survival of patients who underwent neoadjuvant chemotherapy and immunotherapy plus esophagectomy requires further investigation.

In conclusion, esophagectomy following neoadjuvant ICIs plus chemotherapy for patients with resectable ESCC appears to be safe and feasible, with acceptable complications experienced in this small, retrospective study. Complete resection appears to be feasible in most cases, and 30-day and 90-day mortality and 30-day readministration were reasonable during follow-up. We anticipate that the data from ongoing clinical trials will provide high-quality evidence on safety and efficacy in the future.

## Data Availability

The original contributions presented in the study are included in the article/Supplementary Material, further inquiries can be directed to the corresponding author/s.
